# Age-Associated Variation in Brainstem Anteroposterior Dimensions on 3-Tesla MRI: A Single-Center Study of an Imaging-Normal Clinical Cohort

**DOI:** 10.7759/cureus.117899

**Published:** 2026-10-14

**Authors:** Ajay Chand P, Melisha Dsouza, Sam J Mathew, Linta Teresa Tom, Bibin Joseph, Eldhos Jacob

**Affiliations:** 1 Department of Medical Imaging Technology, Father Muller College of Allied Health Sciences, Mangalore, IND; 2 Department of Medical Imaging Technology, AlSharq Hospital, Fujairah, ARE; 3 Department of Medical Imaging Technology, Faculty of Physiotherapy and Allied Health Sciences, M.S. Ramaiah University of Applied Sciences, Bangalore, IND; 4 Department of Respiratory Medicine, Father Muller Medical College Hospital, Mangalore, IND

**Keywords:** aging, brainstem, brainstem dimensions, magnetic resonance imaging, medulla, midbrain, morphometry, pons

## Abstract

Introduction: Brainstem morphometry on magnetic resonance imaging (MRI) provides quantitative information on age-related structural variation. Although previous studies have reported brainstem dimensions in different populations, the relationship between age and anteroposterior (AP) brainstem dimensions remains incompletely characterized in imaging-normal clinical cohorts. This study evaluated age- and sex-related variation in the AP diameters of the midbrain, pons, and medulla in adults undergoing brain MRI.

Methods: This retrospective cross-sectional study included 140 adults (70 males and 70 females) aged ≥18 years who underwent brain MRI for non-brainstem-related clinical indications and demonstrated no brainstem abnormalities. MRI examinations were performed using a 3.0-Tesla Philips Ingenia MRI system (Philips Medical Systems Nederland B.V., Best, The Netherlands). AP diameters of the midbrain, pons, and medulla were measured on midsagittal T1-weighted images using electronic calipers. Differences between sexes were assessed using independent-samples t-tests, while differences across five age categories (18-30, 31-40, 41-50, 51-60, and >60 years) were assessed using one-way analysis of variance with post-hoc testing. Age was also analyzed as a continuous variable using regression models adjusted for sex. Statistical analyses were performed using IBM SPSS Statistics for Windows, Version 25 (Released 2017; IBM Corp., Armonk, New York).

Results: The mean AP diameters of the midbrain, pons, and medulla were 16.84 ± 1.50 mm, 22.06 ± 1.45 mm, and 12.74 ± 1.06 mm, respectively. No statistically significant sex differences were observed for the midbrain (t(138) = −1.96, p = 0.052), pons (t(138) = 0.15, p = 0.885), or medulla (t(138) = 1.33, p = 0.185), although a marginal trend was observed for the midbrain (p = 0.052). Midbrain AP diameter differed across age categories (F(4,135) = 7.29, p < 0.001, η² = 0.178), with the smallest mean diameter in participants aged >60 years. Post-hoc analysis showed greater midbrain diameters in the 18-30, 31-40, and 41-50-year groups than in the >60-year group. Pons diameter showed an overall age-group difference (F(4,135) = 2.73, p = 0.032, η² = 0.075), but no individual pairwise comparison was statistically significant. Medulla diameter did not differ significantly across age groups (F(4,135) = 0.41, p = 0.803, η² = 0.012). In sex-adjusted continuous-age regression, midbrain AP diameter showed an inverse association with age (β = −0.036 mm/year, t(137) = −5.23, 95% CI: −0.050 to −0.023, p < 0.001), whereas associations were not statistically significant for the pons (β = −0.002 mm/year, t(137) = −0.27, 95% CI: −0.016 to 0.013, p = 0.793) or medulla (β = −0.005 mm/year, t(137) = −0.98, 95% CI: −0.015 to 0.005, p = 0.333). No statistically significant age-by-sex interactions were identified.

Conclusions: In this single-center study of an imaging-normal clinical cohort, midbrain AP diameter demonstrated an age-associated reduction, whereas the pons and medulla showed no significant linear age association. No statistically significant sex differences or age-by-sex interactions were identified. These findings provide exploratory baseline measurements of brainstem AP dimensions in an imaging-normal clinical cohort and support further investigation using larger multicenter cohorts, standardized MRI protocols, reproducibility assessment, and three-dimensional or automated morphometric approaches.

## Introduction

Magnetic resonance imaging (MRI) provides high soft-tissue contrast and multiplanar visualization, allowing quantitative assessment of brainstem morphology in vivo. The brainstem, comprising the midbrain, pons, and medulla, contains critical neural pathways and nuclei involved in motor, autonomic, respiratory, and other essential physiological functions [1].

Age-related changes in brain structure are well established, but the magnitude and distribution of these changes may differ among brain regions. In an Indian cohort, Ranganath et al. evaluated age- and sex-related measurements of the midbrain, pons, and medulla in healthy adults and reported age-associated variation in brainstem dimensions [2]. Subsequent MRI-based investigations have similarly examined age-related changes and reference measurements of brainstem structures across different populations [3,4]. These findings suggest that individual components of the brainstem may demonstrate different patterns of structural variation with advancing age.

The midbrain, in particular, has been investigated in quantitative neuroimaging because its morphology can demonstrate measurable variation across age groups and study populations [5]. Quantitative assessment of the pons and medulla may also provide useful reference information when evaluating structural abnormalities of the brainstem. Sex-related differences in brainstem morphology have additionally been investigated, although the magnitude and consistency of such differences vary among studies [6].

Recent developments in quantitative neuroimaging have expanded the assessment of age- and sex-related brain morphology beyond conventional linear measurements. MRI-based volumetric and segmentation approaches have been used to characterize regional brain morphology and quantify structural variation across different age groups [7]. Quantitative MRI approaches have also been applied to age-stratified assessment of brain structures in Indian populations, demonstrating the potential value of population-specific imaging datasets [8]. Previous MRI-based morphometric work has specifically examined brainstem dimensions across a broad age range, demonstrating that the mesencephalon, pons, and medulla may show different patterns of age-related change [9]. Previous MRI-based morphometric studies have also investigated structural variation of the brainstem across different age groups [10]. Although these approaches differ from the linear brainstem measurements used in the present study, they highlight the broader potential of quantitative MRI for characterizing structural variation and provide a rationale for future studies incorporating volumetric, automated, and microstructural assessment of the brainstem.

Differences in study populations, MRI acquisition protocols, measurement landmarks, age distributions, and analytical approaches can contribute to variation in reported brainstem dimensions. In addition, measurements obtained from healthy community-based populations may differ from those obtained from clinically referred individuals undergoing MRI for unrelated indications. Therefore, reference measurements should be interpreted in the context of the population from which they are derived rather than being considered universally applicable normative values.

The present study was designed to evaluate the anteroposterior (AP) diameters of the midbrain, pons, and medulla on midsagittal T1-weighted MRI in adults with imaging-normal brain examinations. Rather than establishing population-wide normative values, this study provides exploratory baseline measurements derived from a single-center clinical cohort undergoing MRI for non-brainstem-related indications. We specifically examined the association between brainstem AP dimensions and chronological age and evaluated whether these measurements differed according to biological sex.

Understanding age-associated variation in brainstem dimensions may provide useful reference information when interpreting brainstem morphology in routine clinical imaging. It may also provide a basis for future investigations using larger multicenter cohorts, standardized MRI protocols, formal measurement-reproducibility assessment, volumetric or automated segmentation, and three-dimensional morphometric approaches.

## Materials and methods

Study design and setting

This retrospective cross-sectional study was conducted between July 2025 and January 2026 in the Department of Radiodiagnosis, Father Muller Medical College Hospital, Mangaluru, Karnataka, India. The study included adults referred for brain MRI for non-brainstem-related clinical indications whose MRI examinations showed no brainstem abnormalities. MRI examinations were performed using a 3.0-Tesla Philips Ingenia CX MRI system (Philips Medical Systems Nederland B.V., Best, The Netherlands). The study was approved by the Father Muller Institutional Ethics Committee (FMIEC; Ref. No. FMIEC/CCM/645/2025; Protocol No. 714/2025; approval date: July 17, 2025).

Study demographics

The source population comprised adults aged 18 years and above who underwent brain MRI for non-brainstem-related clinical indications at the Department of Radiodiagnosis, Father Muller Medical College Hospital, Mangaluru, Karnataka, India, between July 2025 and January 2026. The study included 140 eligible participants whose MRI examinations demonstrated no brainstem abnormalities, comprising 70 males and 70 females. All eligible MRI records meeting the predefined inclusion and exclusion criteria during the study period were included using consecutive sampling. Accordingly, the study represents an imaging-normal clinical cohort from a single center and should not be considered a population-based or regionally representative sample.

The inclusion criteria were age ≥18 years, brain MRI performed for a non-brainstem-related clinical indication, and the absence of brainstem abnormalities on MRI. The exclusion criteria were age <18 years, any brainstem abnormality on MRI, previous neurosurgical intervention, or inadequate image quality for measurement.

MRI acquisition protocol

All MRI examinations were performed using a 3-Tesla Philips Ingenia CX scanner. Brainstem measurements were obtained from midsagittal T1-weighted images. The documented acquisition parameters included a repetition time (TR) of 400 ms, an echo time (TE) of 10 ms, a field of view (FOV) of 230 mm, and a slice thickness of 5 mm. The acquisition matrix, in-plane resolution, flip angle, number of signal averages, and other sequence-specific parameters were not available in the study records and therefore could not be reported. The measurements were performed using electronic calipers in the Philips IntelliSpace Portal.

The in-plane pixel dimension could not be calculated because the acquisition matrix and pixel spacing were unavailable in the study records. The 5-mm slice thickness represents the through-plane dimension and does not establish the in-plane pixel size or the minimum reliably measurable difference. As measurement reproducibility and minimum detectable change were not assessed, the smallest reliably measurable difference could not be determined. Accordingly, the observed age-related association should be interpreted as a statistical finding, with caution regarding its measurement precision and clinical significance.

Brainstem morphometric measurements

The AP diameters of the midbrain, pons, and medulla were measured on midsagittal T1-weighted MRI images using electronic calipers in Philips IntelliSpace Portal. The midbrain AP diameter was measured at the intercollicular level, between the superior and inferior colliculi, extending anteriorly to the interpeduncular fossa. The pons AP diameter was measured at the level of maximal anterior convexity, perpendicular to the floor of the fourth ventricle. The medulla AP diameter was measured at the open medulla, at the lower fourth-ventricle level, between the anterior medullary surface and the posterior boundary near the obex. Measurements were recorded in millimeters, and the corresponding anatomical locations are illustrated in Figure 1.

**Figure 1:**
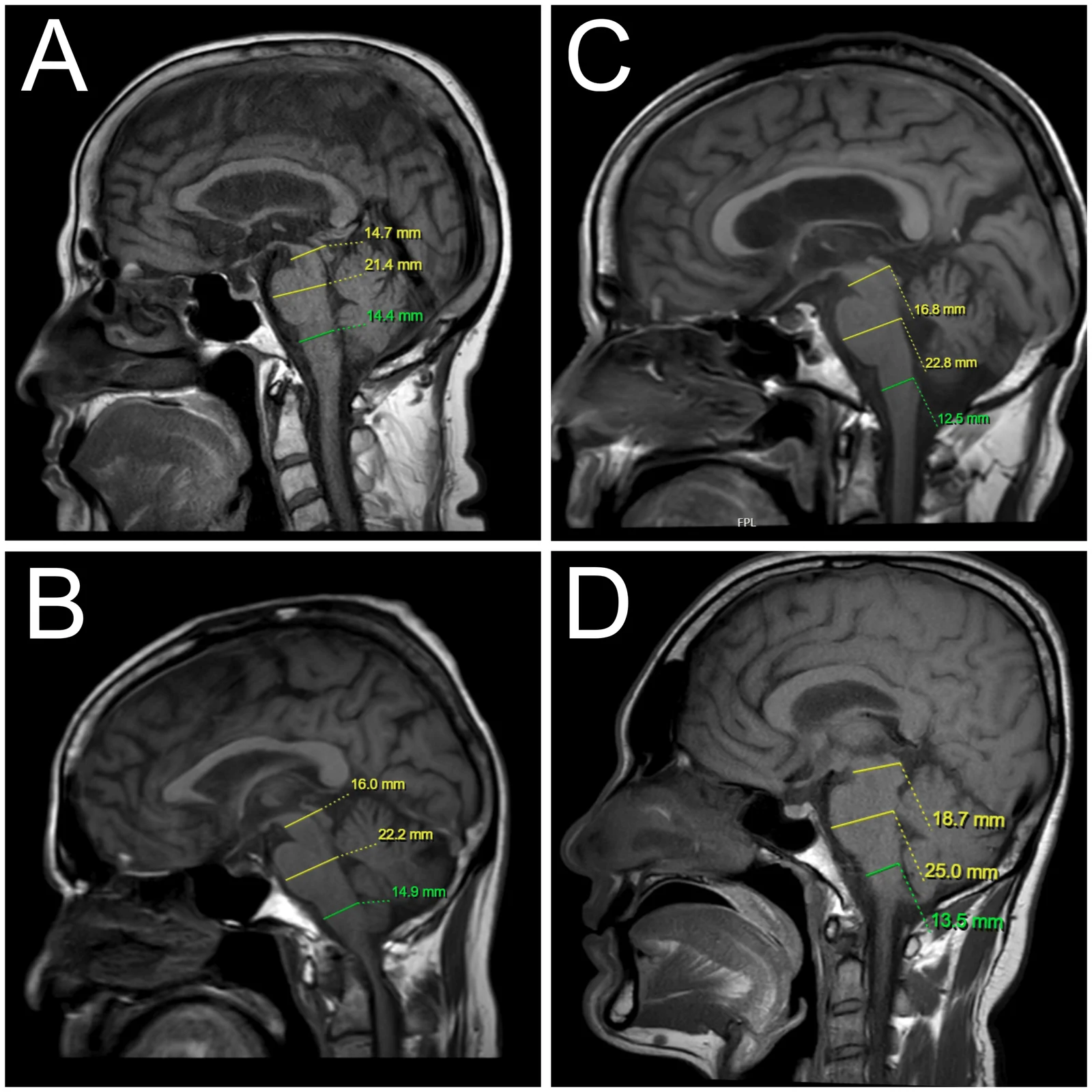
Representative Midsagittal MRI Images Illustrating Brainstem Morphometric Measurements Representative midsagittal T1-weighted MRI images (A–D) illustrating the anteroposterior (AP) measurements of the brainstem. The upper yellow line indicates the midbrain AP diameter measured at the intercollicular level, extending from the intercollicular notch to the interpeduncular fossa. The middle yellow line indicates the maximal pontine AP diameter. The lower green line indicates the medulla oblongata AP diameter measured at the open medulla, at the level of the fourth ventricle. All measurements are expressed in millimeters.

Age classification

For descriptive and categorical analyses, participants were classified into five age groups: 18-30, 31-40, 41-50, 51-60, and >60 years. Chronological age was also analyzed as a continuous variable in the regression analyses.

Sample size

The sample size was calculated using a comparison of two means based on the AP diameter of the pons. The assumed standard deviations were 0.18 cm and 0.17 cm, with a mean difference of 0.10 cm, a two-sided alpha error of 5%, and 80% statistical power. The sample size was calculated using the following formula:

$n = \frac{\left(Z_{\alpha/2} + Z_{\beta}\right)^2\left(\sigma_1^2 + \sigma_2^2\right)}{\left(\mu_1 - \mu_2\right)^2}$,

where Zα/2 = 1.96, Zβ = 0.84, σ₁ = 0.18 cm, σ₂ = 0.17 cm, and μ₁ − μ₂ = 0.10 cm. The calculated minimum sample size was 48.06 participants per group, which was rounded up to 49 participants per group. The final study sample comprised 140 participants, including 70 males and 70 females, exceeding the calculated minimum for the two-group comparison. This calculation was based on the pons AP diameter comparison and was not specifically designed to determine the sample size required for the age-group analyses or age-by-sex interaction.

Statistical analysis

Continuous variables were summarized using means and standard deviations, while categorical variables were summarized using frequencies and percentages. Differences in brainstem AP diameters between males and females were assessed using independent-samples t-tests. Comparisons across the five predefined age groups were performed using one-way analysis of variance (ANOVA). When the overall ANOVA was statistically significant, post-hoc pairwise comparisons were conducted using Tukey’s honestly significant difference (HSD) test, with adjusted p-values reported.

Chronological age was also analyzed as a continuous variable. Separate multiple linear regression models were fitted for the AP diameters of the midbrain, pons, and medulla, with chronological age as the primary predictor and sex as a covariate. Age-by-sex interaction terms were additionally evaluated to determine whether the association between age and brainstem AP diameter differed between males and females. Regression coefficients (β), standard errors, t-statistics, degrees of freedom, 95% confidence intervals, and p-values were reported.

Statistical analyses were performed using IBM SPSS Statistics for Windows, Version 25 (Released 2017; IBM Corp., Armonk, New York). A two-sided p-value <0.05 was considered statistically significant.

## Results

Study demographic

A total of 140 adults were included in the analysis, comprising 70 males and 70 females. The mean age of the study population was 49.81 ± 17.04 years (range: 18-84 years). Participants were distributed across five age groups: 23 (16.4%) were aged 18-30 years, 20 (14.3%) were aged 31-40 years, 26 (18.6%) were aged 41-50 years, 30 (21.4%) were aged 51-60 years, and 41 (29.3%) were aged >60 years. The demographic characteristics of the study population are summarized in Table 1.

**Table 1:** Demographic characteristics of the study population SD, standard deviation.

Characteristic	Overall (N=140)
Total participants, n	140
Male, n (%)	70 (50.0%)
Female, n (%)	70 (50.0%)
Age, mean ± SD (years)	49.81 ± 17.04
Age range (years)	18–84

Overall brainstem measurements

The mean AP diameter of the midbrain was 16.84 ± 1.50 mm (range: 12.5-23.7 mm). The mean AP diameter of the pons was 22.06 ± 1.45 mm (range: 18.8-27.4 mm), while the mean AP diameter of the medulla was 12.74 ± 1.06 mm (range: 10.7-15.9 mm).

Brainstem measurements across age groups

The AP diameters of the midbrain, pons, and medulla across the five predefined age groups are presented in Table 2. The mean midbrain AP diameter was 17.58 ± 1.70 mm in the 18-30-year group, 17.56 ± 1.31 mm in the 31-40-year group, 17.07 ± 1.44 mm in the 41-50-year group, 16.77 ± 1.32 mm in the 51-60-year group, and 15.97 ± 1.23 mm among participants aged >60 years.

**Table 2:** Brainstem anteroposterior dimensions across age groups Values are presented as mean ± SD. Age groups were predefined as 18–30, 31–40, 41–50, 51–60, and >60 years. AP, anteroposterior; SD, standard deviation.

Age group	n	Midbrain AP (mm), mean ± SD	Pons AP (mm), mean ± SD	Medulla AP (mm), mean ± SD
18–30	23	17.58 ± 1.70	21.54 ± 1.20	12.71 ± 0.74
31–40	20	17.56 ± 1.31	22.13 ± 1.68	12.89 ± 0.99
41–50	26	17.07 ± 1.44	22.64 ± 1.58	12.85 ± 1.24
51–60	30	16.77 ± 1.32	22.34 ± 1.12	12.80 ± 1.17
>60	41	15.97 ± 1.23	21.73 ± 1.48	12.58 ± 1.05

The mean AP diameter of the medulla was 12.71 ± 0.74 mm, 12.89 ± 0.99 mm, 12.85 ± 1.24 mm, 12.80 ± 1.17 mm, and 12.58 ± 1.05 mm in the 18-30, 31-40, 41-50, 51-60, and >60-year age groups, respectively. One-way ANOVA revealed no statistically significant difference in medulla AP diameter across the five age groups (F(4,135) = 0.41, p = 0.803, η² = 0.012; Table 3).

**Table 3:** One-way analysis of variance comparing brainstem anteroposterior dimensions across age groups One-way analysis of variance (ANOVA) was used to compare brainstem AP diameters across the five age groups. Statistical significance was defined as p < 0.05. AP, anteroposterior; F, ANOVA F-statistic; df, degrees of freedom; η², eta-squared effect size.

Outcome	F-statistic (df)	p-value	η²
Midbrain AP diameter	7.29 (4,135)	<0.001	0.178
Pons AP diameter	2.73 (4,135)	0.032	0.075
Medulla AP diameter	0.41 (4,135)	0.803	0.012

One-way ANOVA demonstrated a statistically significant difference in midbrain AP diameter across the five age groups (F(4,135) = 7.29, p < 0.001, η² = 0.178). Tukey’s HSD post-hoc analysis revealed that the 18-30, 31-40, and 41-50-year groups had significantly greater mean midbrain AP diameters than the >60-year group, with mean differences of 1.61, 1.59, and 1.11 mm, respectively (adjusted p < 0.001, <0.001, and 0.015, respectively; Table 4).

**Table 4:** Post-hoc pairwise comparisons of midbrain AP diameter across age groups Tukey’s honestly significant difference (HSD) post-hoc test was performed following one-way ANOVA. Mean differences are expressed in millimeters. Adjusted p-values <0.05 were considered statistically significant.

Age-group comparison	Mean difference (mm)	Tukey HSD adjusted p-value
18–30 vs. >60 years	1.61	<0.001
31–40 vs. >60 years	1.59	<0.001
41–50 vs. >60 years	1.11	0.015

The mean pons AP diameter was 21.54 ± 1.20 mm, 22.13 ± 1.68 mm, 22.64 ± 1.58 mm, 22.34 ± 1.12 mm, and 21.73 ± 1.48 mm in the 18-30, 31-40, 41-50, 51-60, and >60-year groups, respectively. One-way ANOVA demonstrated an overall statistically significant difference across age groups (F(4,135) = 2.73, p = 0.032, η² = 0.075). However, Tukey HSD post-hoc analysis did not identify statistically significant differences between individual age groups (Tables 2, 3).

The mean medulla AP diameter was 12.71 ± 0.74 mm, 12.89 ± 0.99 mm, 12.85 ± 1.24 mm, 12.80 ± 1.17 mm, and 12.58 ± 1.05 mm across the five age groups, respectively. No statistically significant difference was observed across age groups (F(4,135) = 0.41, p = 0.803, η² = 0.012; Tables 2, 3).

The distributions of midbrain, pons, and medulla AP diameters across the five age groups are illustrated in Figures 2A-C, respectively.

**Figure 2:**
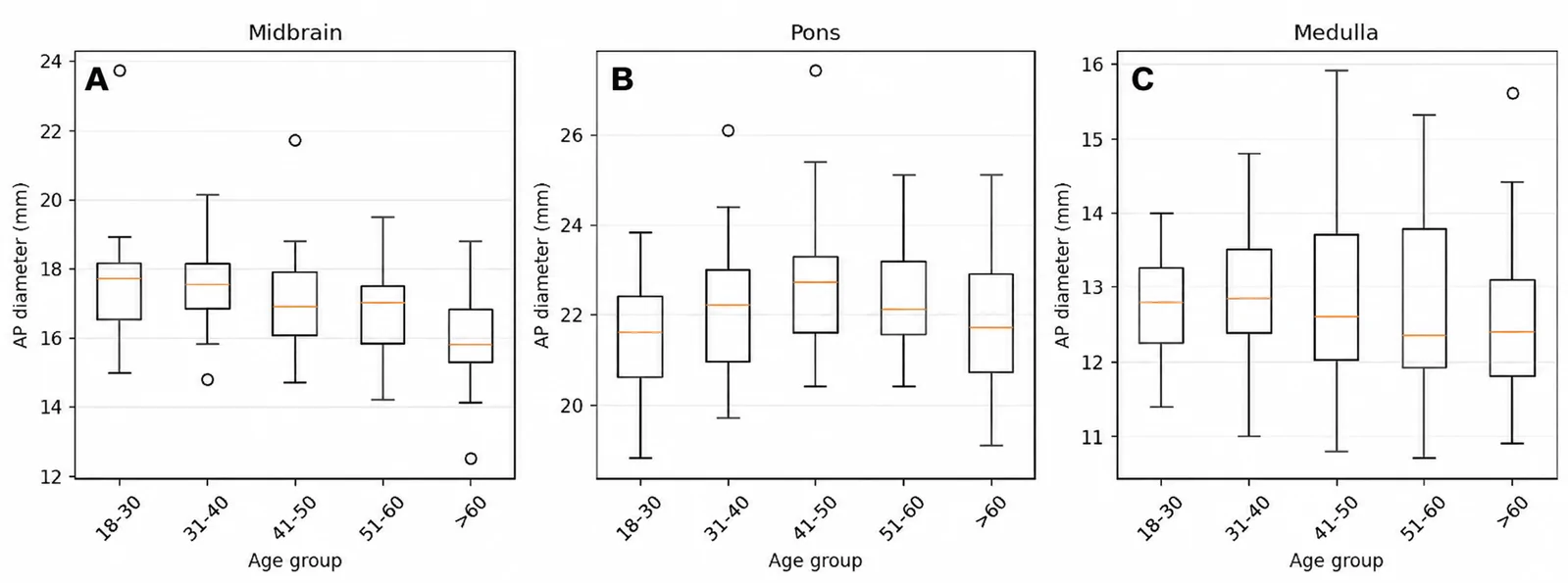
Distribution of Brainstem Anteroposterior Diameters Across Age Groups Box-and-whisker plots showing the distribution of anteroposterior (AP) diameters across five age groups (18–30, 31–40, 41–50, 51–60, and >60 years). (A) Midbrain AP diameter, (B) pons AP diameter, and (C) medulla AP diameter. The horizontal line within each box represents the median, while the lower and upper edges of the box indicate the 25th and 75th percentiles, respectively, defining the interquartile range (IQR). Whiskers indicate the data spread according to the plotting convention used, and individual circles represent outliers.

Sex-related differences

The comparison of brainstem AP diameters between males and females is presented in Table 5. The mean midbrain AP diameter was 16.59 ± 1.35 mm in males and 17.08 ± 1.61 mm in females, with no statistically significant difference between the groups (t(138) = −1.96, p = 0.052). The mean pons AP diameter was 22.07 ± 1.52 mm in males and 22.04 ± 1.39 mm in females (t(138) = 0.15, p = 0.885). The mean medulla AP diameter was 12.86 ± 1.06 mm in males and 12.62 ± 1.04 mm in females (t(138) = 1.33, p = 0.185). No statistically significant sex differences were observed in the AP diameters of the midbrain, pons, or medulla. However, a marginal trend was observed for the midbrain (p = 0.052), whereas no such trend was evident for the pons (p = 0.885) or medulla (p = 0.185).

**Table 5:** Comparison of brainstem anteroposterior diameters between males and females Values are presented as mean ± standard deviation. Between-sex comparisons were performed using independent-samples t-tests. Degrees of freedom were 138 for all comparisons. Cohen’s d represents the standardized mean difference. AP, anteroposterior; SD, standard deviation.

Measure	Male, mean ± SD (mm)	Female, mean ± SD (mm)	t-statistic (df)	p-value	Cohen’s d
Midbrain	16.59 ± 1.35	17.08 ± 1.61	−1.96 (138)	0.052	−0.33
Pons	22.07 ± 1.52	22.04 ± 1.39	0.15 (138)	0.885	0.03
Medulla	12.86 ± 1.06	12.62 ± 1.04	1.33 (138)	0.185	0.22

Continuous-age regression analysis

The results of the continuous-age regression analysis are summarized in Table 6. After adjustment for sex, midbrain AP diameter demonstrated a significant inverse association with chronological age (β = −0.036 mm/year, t(137) = −5.23, 95% CI: −0.050 to −0.023, p < 0.001).

**Table 6:** Continuous-age regression analysis of brainstem AP diameters β represents the estimated change in AP diameter (mm) for each one-year increase in chronological age. Separate linear regression models were fitted with chronological age as the continuous predictor and sex as a covariate. The age × sex interaction was assessed by including the age × sex interaction term in the corresponding regression model. Interaction statistics are presented as absolute t-statistics. SE, standard error; CI, confidence interval; AP, anteroposterior.

Outcome	β (mm/year)	SE	t-statistic (df)	95% CI	p-value	Age × sex interaction, |t| (df)	Interaction p-value
Midbrain	−0.036	0.0069	−5.23 (137)	−0.050 to −0.023	<0.001	0.083 (136)	0.934
Pons	−0.002	0.0074	−0.27 (137)	−0.016 to 0.013	0.793	1.183 (136)	0.239
Medulla	−0.005	0.0051	−0.98 (137)	−0.015 to 0.005	0.333	0.790 (136)	0.431

No statistically significant linear association was observed between chronological age and pons AP diameter after adjustment for sex (β = −0.002 mm/year, t(137) = −0.27, 95% CI: −0.016 to 0.013, p = 0.793). The relationship between chronological age and pons AP diameter is illustrated in Figure 3.

**Figure 3:**
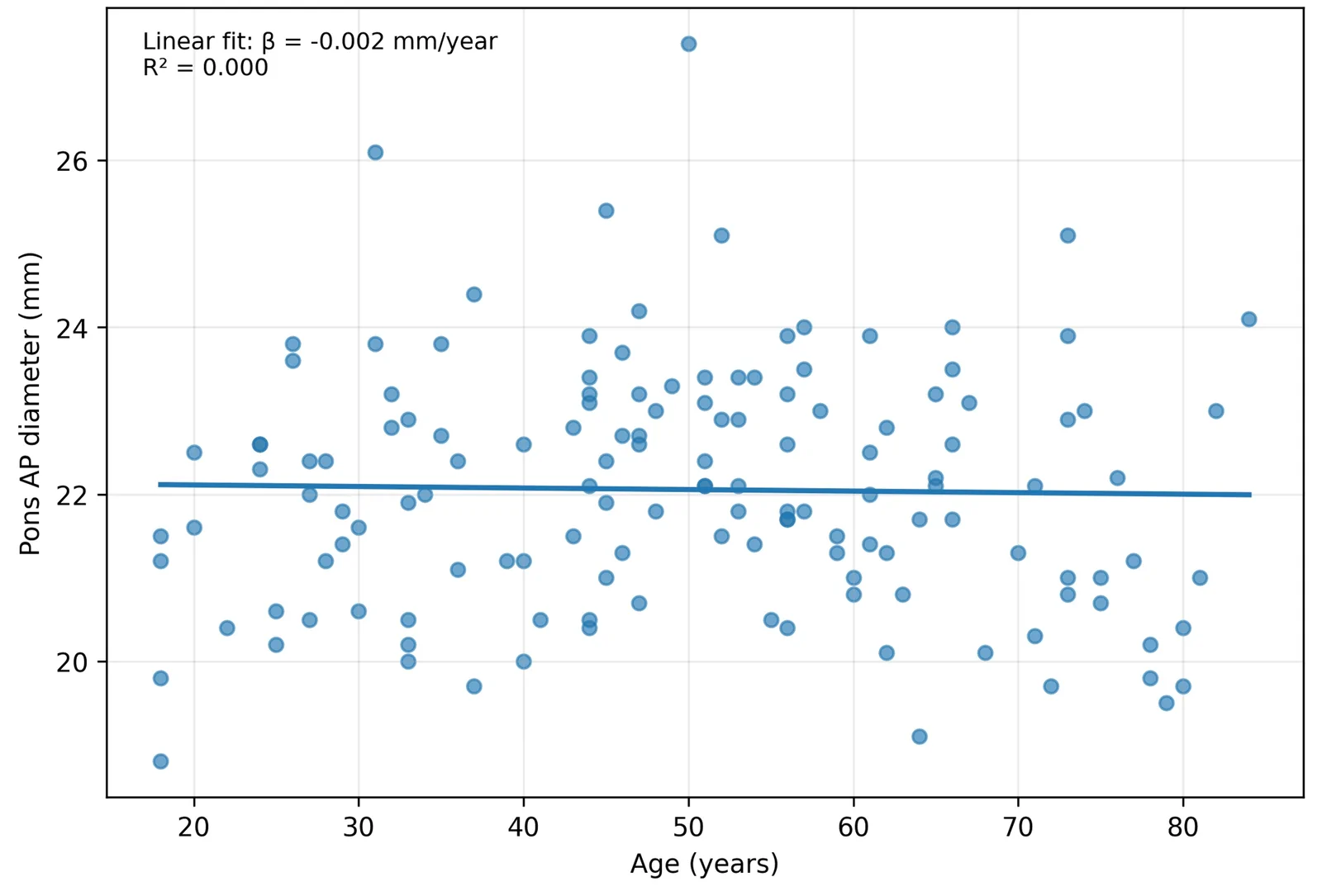
Association Between Age and Pons Anteroposterior Diameter Association between chronological age and pons anteroposterior diameter. Each point represents an individual participant. The fitted line represents the linear association between chronological age and pons anteroposterior diameter.

Similarly, no statistically significant linear association was observed between chronological age and medulla AP diameter after adjustment for sex (β = −0.005 mm/year, t(137) = −0.98, 95% CI: −0.015 to 0.005, p = 0.333). The relationship between chronological age and medulla AP diameter is illustrated in Figure 4.

**Figure 4:**
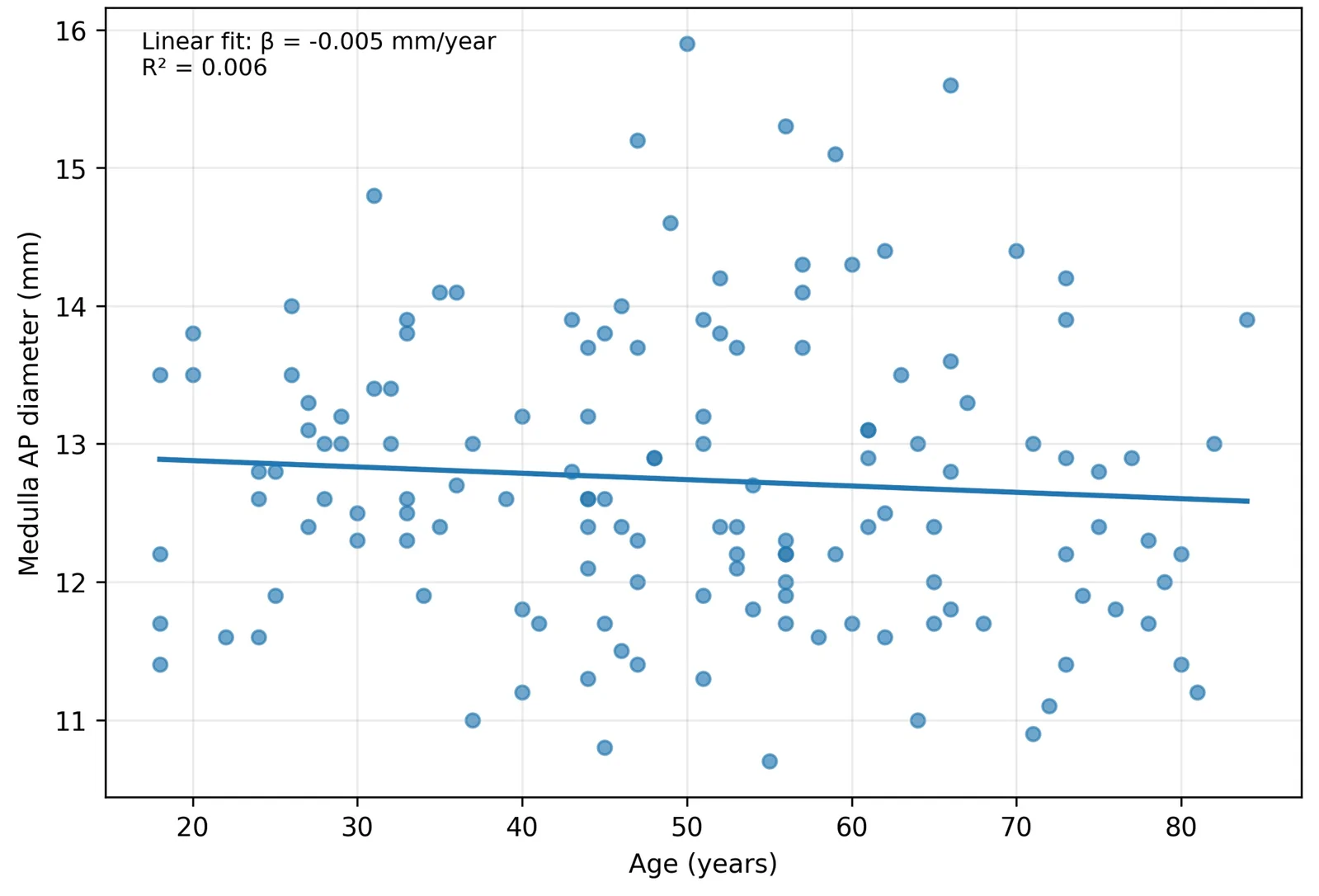
Association Between Age and Medulla Anteroposterior Diameter Association between chronological age and medulla anteroposterior diameter. Each point represents an individual participant. The fitted line represents the linear association between chronological age and medulla anteroposterior diameter.

Age-by-sex interaction

No statistically significant age-by-sex interaction was observed for midbrain AP diameter (t(136) = 0.083, p = 0.934), pons AP diameter (t(136) = 1.183, p = 0.239), or medulla AP diameter (t(136) = 0.790, p = 0.431). These findings indicate that no statistically significant differences in the association between chronological age and brainstem AP dimensions were detected between males and females. The inverse association between chronological age and midbrain AP diameter is illustrated in Figure 5.

**Figure 5:**
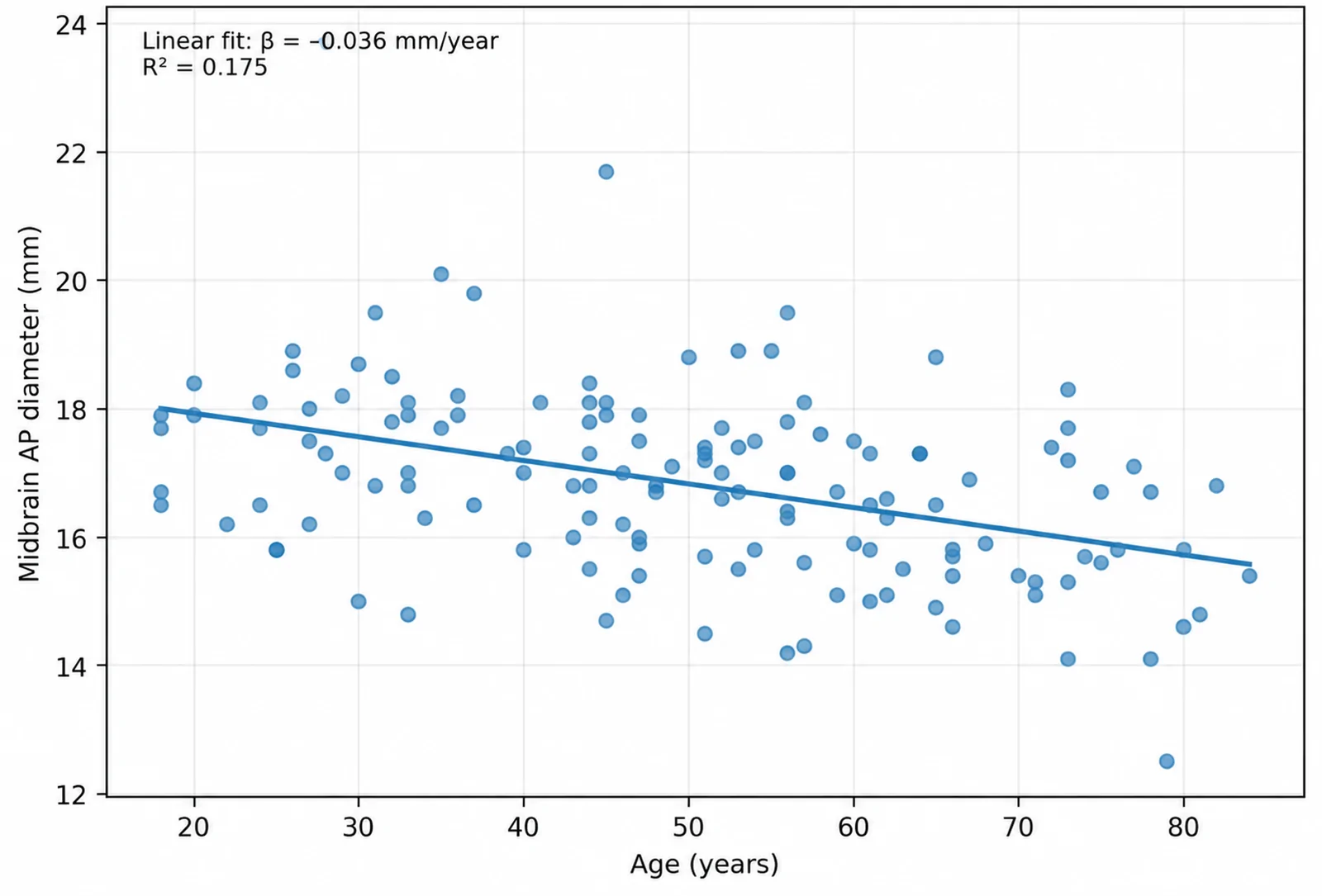
Association Between Age and Midbrain Anteroposterior Diameter Association between chronological age and midbrain anteroposterior diameter. Each point represents an individual participant. The fitted line represents the linear association between chronological age and midbrain anteroposterior diameter.

## Discussion

The present study evaluated age- and sex-related variation in the AP dimensions of the midbrain, pons, and medulla using 3-Tesla MRI in 140 adults with no radiological evidence of brainstem abnormalities. The principal finding was a significant inverse association between chronological age and midbrain AP diameter, whereas no significant continuous age-related association was observed for the pons or medulla. In contrast, sex-related differences were not statistically significant for any of the three brainstem measurements. These findings indicate that age-related morphometric variation is not uniform across the brainstem.

The observed age-related reduction in midbrain AP diameter is consistent with previous MRI-based investigations reporting age-associated changes in brainstem morphology [1,2,4]. Polat et al. evaluated brainstem morphometry in 263 healthy Turkish individuals aged 18-65 years and demonstrated age- and sex-related variation in several brainstem measurements [1]. Similarly, Ranganath et al., in a hospital-based Indian cohort of 142 adults, reported reductions in several brainstem measurements with advancing age, while the medulla at the pontomedullary junction showed relative stability [2]. These findings suggest that age-related changes may vary across different brainstem regions.

In the present study, midbrain AP diameter demonstrated a statistically significant inverse association with chronological age after adjustment for sex (β = −0.036 mm/year, t = −5.23, 95% CI: −0.050 to −0.023, p < 0.001). However, the magnitude of this coefficient requires cautious interpretation. As the study used a cross-sectional design, the coefficient represents an age-associated difference between participants rather than a directly observed annual reduction within an individual. Although the association was statistically significant, its physical detectability and clinical relevance cannot be established from the present data. The 5-mm slice thickness, image resolution, and potential variability in manual caliper placement may affect measurement precision. Furthermore, formal intraobserver and interobserver reliability assessments were not performed, and the minimum detectable difference was not established. Therefore, statistical significance should not be interpreted as evidence of a reliably measurable anatomical change or clinically meaningful atrophy. This finding should be considered exploratory and requires validation using standardized high-resolution MRI protocols, formal measurement-reproducibility assessments, and longitudinal studies.

The findings for the pons were less consistent with those observed for the midbrain. Although comparison across the predefined age groups showed an overall difference (F(4,135) = 2.73, p = 0.032, η² = 0.075), continuous regression did not demonstrate a significant association between chronological age and pons AP diameter (β = −0.002 mm/year, t = −0.27, 95% CI: −0.016 to 0.013, p = 0.793). No individual post-hoc pairwise comparison reached statistical significance. This pattern illustrates the importance of evaluating age as a continuous variable in addition to categorical age-group comparisons and indicates that an overall group-level difference does not necessarily translate into significant pairwise or linear associations.

No significant age-related association was observed for the medulla AP diameter. The estimated change was small and statistically nonsignificant (β = −0.005 mm/year, t = −0.98, 95% CI: −0.015 to 0.005, p = 0.333). Similarly, the age-group analysis did not demonstrate significant differences in medulla AP diameter (F(4,135) = 0.41, p = 0.803, η² = 0.012). This relative stability is consistent with observations from the Indian study by Ranganath et al., in which the medulla at the pontomedullary junction did not demonstrate the same age-related reduction observed at other brainstem levels [2].

No statistically significant differences were identified between males and females for midbrain, pons, or medulla AP diameters. However, a marginal trend was observed for the midbrain (t(138) = −1.96, p = 0.052), although this difference did not reach statistical significance. The pons (t(138) = 0.15, p = 0.885) and medulla (t(138) = 1.33, p = 0.185) showed no statistically significant sex-related differences. These findings are broadly comparable with the Indian study by Ranganath et al., which also reported no statistically significant gender differences in sagittal diameters of the midbrain, pons, and medulla [2]. However, sex-related differences have not been completely uniform across the literature. Oguro et al. reported sex-specific patterns of age-associated brainstem changes in 152 individuals over 40 years of age, with significant age-associated atrophy in selected midbrain and pontine regions, particularly among men [6]. Differences in measurement landmarks, age distribution, sample characteristics, MRI protocols, and analytical methods may contribute to these variations.

The present study should also be interpreted in relation to previously published Indian data. Ranganath et al. evaluated age- and sex-related morphometric measurements of the brainstem using MRI in healthy Indian adults [2]. The present investigation similarly examines linear brainstem dimensions but was conducted in a different single-center clinical cohort using a specific 3-Tesla MRI acquisition and measurement approach. Consequently, the present findings provide additional measurements from an imaging-normal clinical population rather than establishing an independent population-wide normative dataset. Differences between studies should therefore be interpreted cautiously, as variations in participant selection, age distribution, imaging protocols, anatomical landmarks, and measurement procedures may affect the reported dimensions [1,2,4].

The distinction between a clinically referred imaging-normal cohort and a community-based healthy population is important when interpreting these findings. Participants in the present study underwent MRI for clinical indications unrelated to the brainstem and had no radiological evidence of brainstem abnormalities. Although this approach allowed assessment of brainstem dimensions in individuals without apparent brainstem pathology, the cohort may differ from a truly population-based healthy sample. Systemic metabolic conditions, vascular risk factors, cardiovascular health, and other clinical characteristics were not comprehensively incorporated into the present analysis. Such factors may influence brain structure and could potentially contribute to between-individual variation in morphometric measurements. Accordingly, the measurements reported here should be considered reference measurements derived from an imaging-normal clinical cohort rather than definitive normative values for the general Indian population.

The methodological approach also has implications for interpretation. The present study used manual linear AP measurements obtained on midsagittal T1-weighted MRI. Linear measurements have the advantage of being relatively straightforward and potentially applicable to routine clinical imaging. Previous MRI studies have similarly demonstrated that brainstem measurements can be obtained using linear morphometric techniques [1,2,4]. However, linear measurements represent only a limited aspect of three-dimensional brainstem morphology. Brainstem shape, volume, regional tissue composition, and microstructural characteristics cannot be captured by a single AP diameter.

Recent advances in quantitative neuroimaging provide opportunities to extend this approach. Volumetric, segmentation-based, diffusion-sensitive, and other quantitative MRI methods can provide complementary information beyond conventional linear dimensions [7,8,10]. These approaches may help determine whether the age-associated pattern observed for midbrain AP diameter is accompanied by changes in regional volume, shape, tissue microstructure, or other quantitative imaging biomarkers. Such multimodal assessment may be particularly useful in future studies seeking to distinguish normal age-related variation from disease-associated structural changes.

The importance of accounting for individual differences in head size and overall brain volume is also highlighted by the study of Syed Nasser et al. [8], which reported that normalized brain-volume fractions, including brain parenchymal fraction (BPF), gray matter fraction (GMF), and white matter fraction (WMF), may help mitigate sex- and population-related differences in normative imaging curves by accounting for individual variation in head size or brain volume. This approach may have relevance to future brainstem morphometric research, as absolute linear measurements may be influenced by anatomical variability between individuals. However, normalized whole-brain volumetric fractions are not directly equivalent to the regional AP diameters assessed in the present study. Future research could investigate whether brainstem-specific volumetric measurements or appropriately normalized morphometric indices improve comparability across individuals, sexes, and populations.

The clinical relevance of quantitative brainstem morphometry should similarly be considered within the scope of the present study. Brainstem dimensions can provide anatomical reference information when evaluating structural abnormalities. However, the present study was not designed to assess diagnostic accuracy, disease classification, or prognostic performance. In particular, the study does not establish diagnostic thresholds for Parkinsonian disorders, multiple system atrophy, progressive supranuclear palsy, or other neurological diseases. Previous MRI studies have demonstrated disease-associated alterations in brainstem morphometry, including reduced midbrain dimensions in progressive supranuclear palsy [11]. However, extrapolation from an imaging-normal cohort to disease diagnosis would require separate studies involving appropriately characterized patient and control groups.

Limitations

Several limitations should be considered when interpreting the findings of this study. First, the retrospective cross-sectional design does not permit the assessment of longitudinal changes within individual participants. The observed association between chronological age and midbrain AP diameter therefore represents differences between individuals of different ages rather than directly demonstrating progressive structural change over time. Second, the study was conducted at a single institution using a single 3-Tesla MRI system, which may limit the generalizability of the findings to other populations and imaging platforms. Third, the sample-size calculation was based on a two-group comparison of pons AP diameter and was not specifically designed for the multiple age-group comparisons or age-by-sex interaction analyses. Although the final sample of 140 participants exceeded the calculated minimum of 49 participants per group for the original comparison, the study may have had limited statistical power to detect smaller effects or interaction differences. In addition, the specific published source supporting the assumed standard deviations and mean difference used in the original sample-size calculation could not be independently verified.

Fourth, the study relied on manual linear AP measurements rather than three-dimensional volumetric or automated morphometric techniques. Although all examinations were performed using a 3-Tesla MRI system, the acquisition matrix and in-plane pixel spacing for the T1-weighted sequence were unavailable in the study records. Consequently, the physical equivalent of one pixel and the minimum reliably measurable difference could not be determined. The 5-mm slice thickness represents the through-plane dimension and may contribute to partial-volume effects; however, it does not independently establish in-plane measurement precision. Furthermore, formal intraobserver and interobserver reproducibility assessments, measurement error, and minimum detectable change were not evaluated. Therefore, the precision and reproducibility of small differences in brainstem AP dimensions cannot be established from this dataset. The statistically significant inverse association between age and midbrain AP diameter should consequently be interpreted as a cross-sectional statistical association rather than evidence of reliably detectable individual-level anatomical change or clinically meaningful atrophy. Fifth, detailed systemic metabolic and vascular characteristics were not incorporated into the statistical analysis, and their potential contribution to variation in brainstem dimensions could not be assessed. Sixth, participants were clinically referred for MRI rather than recruited from a community-based healthy population. Despite the absence of radiological brainstem abnormalities, this imaging-normal clinical cohort may not represent the general healthy population; therefore, the measurements should be interpreted as cohort-specific reference measurements rather than definitive population-wide normative values.

Despite these limitations, the study provides a structured assessment of brainstem AP dimensions across adulthood and demonstrates the value of combining categorical age-group comparisons with continuous regression analysis. The continuous analysis showed that the relationship between age and brainstem morphology was most evident for the midbrain, while the pons and medulla did not demonstrate significant linear age associations. The absence of significant age-by-sex interactions further suggests that the observed relationships between age and the measured brainstem dimensions did not significantly differ between males and females within this sample.

Future research should involve larger, multicenter cohorts with broader demographic and clinical representation. Standardized MRI acquisition protocols and clearly defined anatomical measurement landmarks would improve comparability between studies. Formal intraobserver and interobserver reproducibility assessment should also be incorporated to establish the reliability of manual measurements. Three-dimensional volumetric analysis and automated or semi-automated segmentation could provide a more comprehensive characterization of brainstem morphology. In addition, incorporation of vascular and metabolic risk factors would allow assessment of potential clinical contributors to structural variation. Longitudinal studies would be particularly valuable for determining whether the age-related reduction in midbrain AP diameter observed in the present cross-sectional analysis corresponds to progressive structural change within individuals.

Overall, the findings indicate that brainstem AP dimensions demonstrate structure-specific patterns of age-related variation in this imaging-normal clinical cohort. The midbrain showed a significant inverse association with chronological age, whereas the pons and medulla did not demonstrate significant continuous age-related associations. No statistically significant sex differences or age-by-sex interactions were identified. These findings provide exploratory baseline measurements of brainstem AP dimensions in an imaging-normal clinical cohort and support further investigation using larger, longitudinal, multicenter, and three-dimensional quantitative imaging approaches.

## Conclusions

In this single-center study of an imaging-normal clinical cohort, midbrain AP diameter demonstrated a significant inverse association with increasing chronological age, whereas no significant continuous age-related associations were observed for the pons or medulla. No statistically significant sex differences or age-by-sex interactions were identified. These findings provide exploratory baseline measurements of brainstem AP dimensions in an imaging-normal clinical cohort and support further investigation using larger multicenter cohorts, standardized MRI protocols, formal intraobserver and interobserver reproducibility assessment, and three-dimensional morphometric approaches to establish more generalizable reference ranges.
